# Relationship Between Frontal QRS-T Angle and Non-Alcoholic Fatty Liver Disease (NAFLD) Fibrosis Score in Patients with Stable Angina Pectoris

**DOI:** 10.3390/jcm14145117

**Published:** 2025-07-18

**Authors:** Ali Gökhan Özyıldız, Afag Özyıldız, Hüseyin Durak, Nadir Emlek, Mustafa Çetin

**Affiliations:** Cardiology Department, Training and Research Hospital, Recep Tayyip Erdoğan University, 53020 Rize, Türkiye; drafagj@gmail.com (A.Ö.); huseyin.durak@erdogan.edu.tr (H.D.); nadir.emlek@erdogan.edu.tr (N.E.); mustafa.cetin@erdogan.edu.tr (M.Ç.)

**Keywords:** frontal QRS-T angle, non-alcoholic fatty liver disease, stable angina pectoris

## Abstract

**Aim:** The frontal QRS-T (fQRS-T) angle serves as an electrocardiography indicator that visually represents the disparity between the frontal QRS axis and the T axis. The heterogeneity between cardiac depolarization and repolarization rises with an increase in the fQRS-T angle. Prior research has demonstrated a relationship between the fQRS-T angle and the extent of atherosclerosis, along with the risk of cardiovascular mortality. The non-alcoholic fatty liver disease fibrosis score (NFS) is a non-invasive scoring tool used to quantify the degree of liver fibrosis in individuals with non-alcoholic fatty liver disease (NAFLD). Non-alcoholic fatty liver disease increases the risk of atherosclerotic cardiovascular disease, which can be predicted using the NFS. The objective of this study is to examine the potential correlation between the fQRS-T angle and NFS in patients with stable angina pectoris. **Materials and Methods:** This cross-sectional study included 177 (48 women) non-alcoholic patients who underwent coronary angiography due to stable angina pectoris. Individual NFS values were calculated using clinical and laboratory data. Patients were categorized into two groups based on a NFS threshold value of 0.67. Following a minimum fasting period of 12 h, biochemical laboratory parameters were acquired using a peripheral venous sample, and electrocardiographic data were recorded. **Results:** The univariate logistic regression analysis revealed significant associations between hypertension (*p* = 0.018), coronary artery disease (*p* = 0.014), neutrophil (*p* = 0.024), hemoglobin (*p* = 0.038), and low-density lipoprotein (LDL, *p* = 0.007) with the NFS. The electrocardiographic variables related to the score included the QRS duration (*p* = 0.015), Pmax (*p* = 0.026), QTC interval (*p* = 0.02), and fQRS-T angle (*p* < 0.001). In the multivariate logistic regression analysis, NFS was independently associated with LDL (OR: 0.984, 95% CI: 0.970–0.998, *p* = 0.024) and fQRS-T angle (OR: 3.472, 95% CI: 1.886–6.395, *p* < 0.001). **Conclusions:** The FQRS-T angle may exhibit a distinct correlation with NAFLD. Extensive investigations should validate this link, since the fibrosis score can serve as an effective tool for monitoring patients prior to the onset of clinical symptoms associated with liver fibrosis.

## 1. Introduction

Electrocardiography (ECG) is a cheap, noninvasive, and practical diagnostic tool used to detect cardiovascular diseases. The frontal QRS-T (fQRS-T) angle is an easily obtainable parameter derived from a surface ECG. This angle reflects an absolute difference between ventricular depolarization and repolarization vectors, and indicates myocardial electrical heterogeneity [1,2]. The frontal QRS-T angle reflects the electrical instability of the myocardium and predicts cardiovascular outcomes, particularly in patients with coronary artery disease (CAD) [3]. In fact, it has been reported that the fQRS-T angle of >100° increases the risk of arrhythmic death in the middle-aged general population [4]. Ventricular repolarization abnormality resulting from ischemia causes an increase in the fQRS-T angle. This increase can be detected prior to significant ECG changes [5].

Non-alcoholic fatty liver disease (NAFLD) is the predominant hepatic disorder that can progress to cirrhosis. It is a silent disease with no subclinical laboratory or radiological findings. The disease is defined as a progression of hepatic steatosis in at least 5% of hepatocytes without high alcohol intake. It includes a wide range of liver diseases that differ between two specific histological types: non-alcoholic fatty liver (NAFL), which is generally benign, and non-alcoholic steatohepatitis (NASH), which is more severe [6]. Atherosclerotic cardiovascular disease is the predominant cause of mortality in NAFLD patients. Stable angina pectoris is a slowly advancing variant of atherosclerosis. Numerous studies have demonstrated that NAFLD is a significant risk factor for the progression of atherosclerosis. Obesity is the predominant etiology of NAFLD, with associated conditions such as impaired glucose homeostasis, dyslipidemia, hypertension, and inflammation, which are prevalent in most NAFLD patients. These metabolic risk factors contribute to the accelerated progression of atherosclerosis in individuals with NAFLD [7].

The clinical relevance of fibrosis arises from its correlation with cardiovascular events and mortality rather than its diagnosis. The association between NAFLD and cardiovascular events is thought to result from common pathophysiological mechanisms, including endothelial dysfunction, systemic inflammation, oxidative stress, insulin, and impaired lipid metabolism rather than a direct causal relationship between the two conditions [8]. Cardiovascular risk calculation tools do not incorporate NAFLD. Furthermore, NAFLD follows a silent course and may not be noticed until an increase in liver enzymes or radiological findings is incidentally detected [9]. Consequently, early diagnosis or suspicion of the disease is of vital importance due to its close relationship with high cardiovascular events and mortality. Various scoring systems have been used to assess the degree of fibrosis. The NAFLD fibrosis score (NFS) is one of the most popular tools. A low cut-off value (−1.455) excludes advanced fibrosis, while a high cut-off value (0.676) identifies advanced fibrosis.

To reduce cardiovascular morbidity and mortality, cardiovascular risk assessment is recommended every 1–2 years in NAFLD. The recommendations for NAFLD include the Framingham and atherosclerotic cardiovascular disease risk scores [10]. In clinical practice, there is a need for easily accessible, inexpensive, and easy-to-evaluate screening tests for NAFLD, particularly for predicting cardiovascular risk. When analyzed together, the f-QRST angle and the NFS are likely to be more reliable in pointing to a possible association between atherosclerotic cardiovascular disease and NAFLD. The aim of the current study is to examine the potential correlation between the fQRS-T angle and NFS in patients with stable angina pectoris.

## 2. Methods

Study Design and Population: The cross-sectional, observational study included a total of 177 consecutive patients aged over 18 years of age admitted to the cardiology clinic due to stable angina pectoris and scheduled for coronary angiography. Informed permission in writing was acquired from all participants. The local ethics committee accepted this study in compliance with the Helsinki Declaration. Patients with heart failure, moderate to severe valvular heart disease, atrial fibrillation, paced rhythm, atrioventricular block, and left or right bundle branch block were excluded from the study.

**Figure 1:**
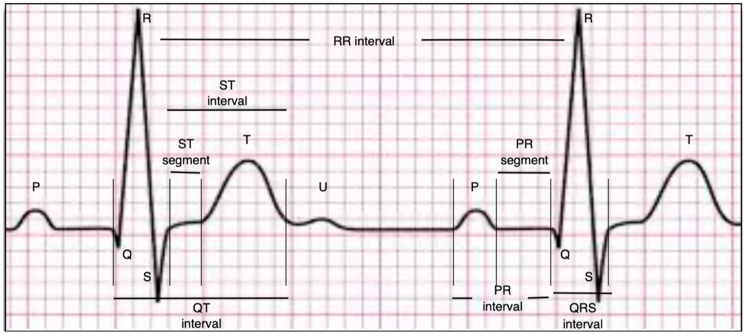
ECG parameters.

**Figure 2:**
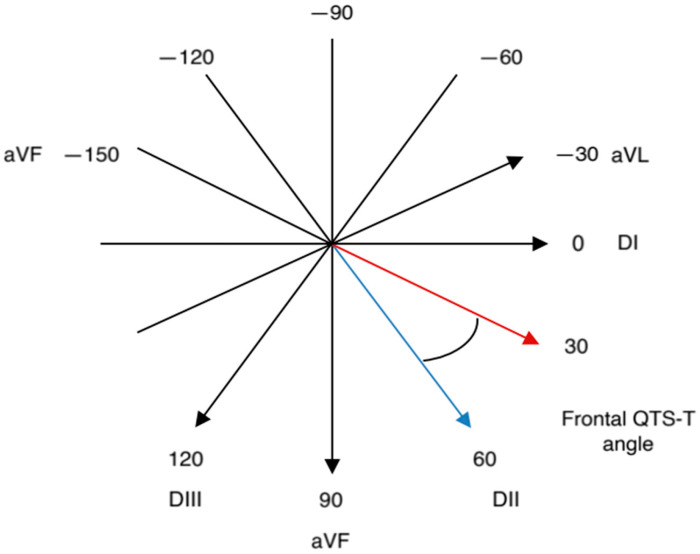
Frontal QRS-T angle: absolute difference between the QRS axis and the T axis.

Demographic characteristics, clinical features, and extensive biochemical parameters of the patients were recorded upon admission. Prior to the coronary angiography procedure, a 12-lead superficial ECG (Nihon Kohden, Tokyo, Japan) was performed in the supine position, with a 25 mm/s paper speed and a voltage of 10 mm/s. Electrocardiograms obtained from patients were scanned and magnified at 300 DPI. Electrocardiography scans were examined for relevant parameters utilizing the online program ‘GeoGebra Software, version 4.2 (International GeoGebra Institute (IGI), Linz, Austria)’. Q-waves over 40 ms in duration and surpassing 25% of the subsequent R or S waves in depth were classified as pathological Q-waves. Fragmented QRS is characterized by extra deflections or notches within the QRS complex, signifying aberrant depolarization patterns. The QRS duration is regarded as the interval from the onset of the Q- or R-wave to the end of the S-wave. P-maximum and P-minimum were calculated by measuring the longest and shortest durations of P-waves from the most apparent leads on the ECG. The P-dispersion was determined by the difference between the specified durations. The QT interval was measured from the onset of the Q-wave to the end of the T-wave. The QTc interval was computed with the Bazett formula (QTc = QT/√RR) in relation to heart rate. The R peak time was defined as the interval from the onset of the QRS complex to the top of the R-wave. The fQRS-T angle was determined from the ECG by calculating the absolute difference between the frontal plane QRS axis and T axis. If the difference exceeds 180°, it is calculated as 360° minus the value of the difference (Figure 1 and Figure 2).

The NAFLD fibrosis score (NFS) estimates the amount of scarring in the liver based on various laboratory tests, including age, glycemia, body mass index (BMI), platelet count, albumin, aspartate aminotransferase (AST), and alanine aminotransferase (ALT). The score uses two cutoff values to exclude advanced fibrosis (>−1.455) or to diagnose advanced fibrosis (<0.676) [11].

Hypertension was defined as blood pressure >140/90 mmHg in repeated measurements or a history of antihypertensive use. Diabetes was defined as fasting blood sugar >125 mg/dL or glucose >200 mg/dL at any measurement or a history of antidiabetic agent use. Dyslipidemia was defined as a total cholesterol value of >200 mg/dL or a history of antilipidemic agent use. Coronary artery disease was defined as having a history of revascularization or angiographically demonstrated >50% stenosis in any coronary artery. Body mass index was calculated using the weight/height^2^ formula. Smoking one cigarette per day was considered a smoking habit.

Statistical analysis: Continuous variables were assessed using visual methods (histograms, probability plots) and analytical techniques (Kolmogorov–Smirnov/Shapiro–Wilk test). Categorical data were presented as percentages, whereas continuous variables were expressed as mean ± standard deviation. The chi-square test or Fisher’s exact test was employed to compare categorical groups, particularly when the assumptions of the chi-square test were not met. Parameters showing a normal distribution were assessed using one-way analysis of variance (ANOVA). Following the comparison of the two groups, the parameters exhibiting statistically significant differences were initially assessed using univariate logistic regression analysis. Subsequently, multivariate logistic regression analysis was conducted utilizing the model established with the parameters that retained significance (shown with *). Sensitivity and specificity were assessed by ROC analysis. Predictive levels for both positive and negative outcomes were computed utilizing the corresponding formulas. A *p*-value of less than 0.05 was deemed statistically significant.

## 3. Results

Patients were divided into two groups according to NFS value. Demographic, clinical, laboratory, and electrocardiographic characteristics of the study groups are shown in Table 1. Of all the patients, 48 (27.1%) were female. The mean age was higher in the group with increased NFS (71.6 vs. 55.8, *p* < 0.001). The prevalence of hypertension (*p* = 0.011), diabetes (*p* = 0.002), and coronary artery disease (CAD) (*p* = 0.002) was higher in this group. Analysis of drug utilization revealed that the use of P2Y12 inhibitors (*p* = 0.042), angiotensin-converting enzyme (ACE) inhibitors (*p* = 0.024), beta-blockers (*p* = 0.029), and oral antidiabetics (*p* = 0.006) was more prevalent in the cohort with elevated NFS. Laboratory findings indicated increased levels of glucose (*p* < 0.001), neutrophils (*p* = 0.021), and AST (*p* < 0.001) in the cohort with a high NFS. Hemoglobin (*p* = 0.034), platelet count (*p* < 0.001), albumin (*p* < 0.001), total cholesterol (*p* = 0.006), and low-density lipoprotein (LDL) cholesterol (*p* = 0.005) levels were higher in the low NFS group. The electrocardiographic measures, including QRS interval (*p* = 0.01), P-max (*p* = 0.019), P-dispersion (*p* = 0.048), QTc interval (*p* = 0.018), and R peak time (*p* = 0.047), were elevated in the cohort with a high NFS. The frontal QRS angle was measured at 24° in the low NFS group and 118° in the high NFS group (*p* < 0.001).

**Figure 3:**
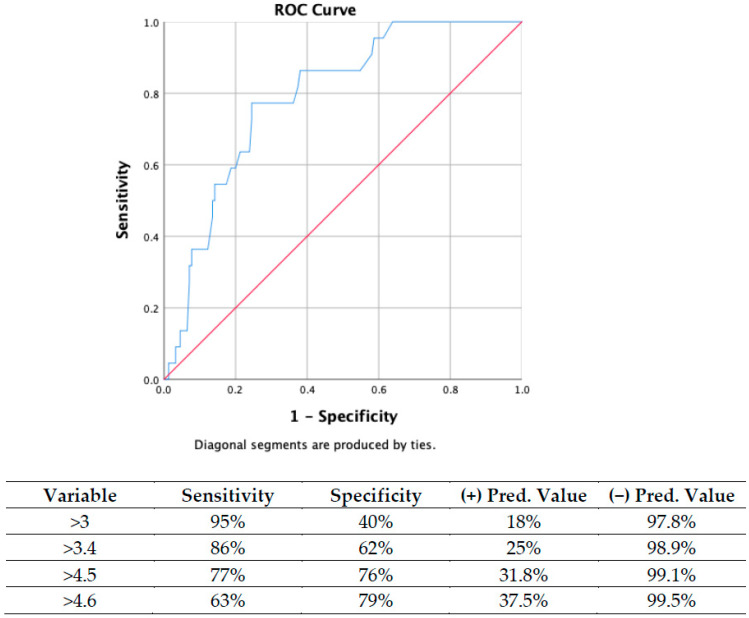
Receiver Operating Characteristic Curve.

The univariate logistic regression analysis revealed significant associations between hypertension (*p* = 0.018), CAD (*p* = 0.014), neutrophil (*p* = 0.024), hemoglobin (*p* = 0.038), and LDL (*p* = 0.007) with the NAFLD fibrosis score. The electrocardiographic variables related to the score included QRS duration (*p* = 0.015), P-max (*p* = 0.026), QTC interval (*p* = 0.02), and the fQRS-T angle (*p* < 0.001) In multivariate logistic regression analysis, an independent relationship was identified between NFS with LDL (OR: 0.984, 95% CI: 0.970–0.998, *p* = 0.024) as well as with the fQRS-T angle (OR: 3.472, 95% CI: 1.886–6.395, *p* < 0.001, Table 2). The predictive value of the fQRS-T angle in patients with a high NFS is illustrated in the ROC analysis (Figure 3).

## 4. Discussion

In the present research, the relationship between the fQRS-T angle and NFS was evaluated. The result of the study demonstrated that the fQRS-T angle had an independent relationship with NFS in patients with stable angina.

Damaged or inhomogeneous myocardium secondary to ischemia results in abnormal ventricular repolarization and consequent widening of the fQRS-T angle. The fQRS-T angle width is a strong predictor of cardiovascular mortality and incidence of CAD. Even in the low-risk population, the fQRS-T angle of >100° is associated with a 3-fold increase in sudden cardiac death. The risk of multivessel disease is significantly higher in patients with a QRS-T angle of >90° compared to those with a QRS-T angle of <90° [12]. Although the spatial QRS-T angle is more consistent than the frontal QRS-T angle in predicting underlying cardiac disease, the fQRS-T angle has the opportunity for widespread use because it is simple to calculate and it depends only on the ECG device [1]. In the current study, the f-QRS-T angle was 118° in the group with NFS ≥ 0.67, supporting the relationship between the f-QRS-T angle and cardiovascular disease risk, as this group exhibited higher rates of advanced age, hypertension, diabetes, and CAD.

Non-alcoholic fatty liver disease is a hepatic steatosis situation in the absence of secondary causes such as viral, hereditary, alcohol consumption, medication, etc. The global prevalence of the disease reaches 30% [13]. Although the precise mechanism for the disease’s augmentation of cardiovascular events remains unclarified, increased arterial stiffness, endothelial dysfunction, and cytokine-mediated inflammatory processes have been proposed as potential contributors [14]. The close relationship between NAFLD and metabolic syndrome further supports this theory. NAFLD shares common risk factors with metabolic syndrome, including insulin resistance, obesity, dyslipidemia, hypertension, and proinflammatory and prothrombotic states [15]. The causal link between CAD and NAFLD remains unclarified; however, several pathophysiological pathways seem to be essential contributors. Insulin resistance, oxidative stress, and atherogenic dyslipidemia induce the generation of proinflammatory, profibrogenic, and vasoactive mediators that yield cardiovascular repercussions. The prevalence of obesity-related metabolic problems among most NAFLD patients substantiates this assertion [16]. NAFLD correlates with a higher prevalence of hypertension. This relationship is presumably mediated by various adverse effects, including inflammation, insulin resistance, activation of the renin–angiotensin–aldosterone system, and stimulation of the sympathetic nervous system. Zhao et al. have shown a close relationship between NAFLD and hypertension [17]. NAFLD may cause diabetes via proinflammatory cytokines by increasing hepatic and peripheral insulin resistance. Our study found that hypertension and diabetes were more prevalent in the cohort with high NFS, consistent with the literature. Dyslipidemia is a prevalent contributor to NAFLD and CAD. The notable prevalence of CAD in the study population contrasts sharply with the low statin usage rates observed in both categories. This low rate can be attributed to the prevalence of medication resistance to statin therapy, both locally and globally. The statin usage rate in our society is 65% following the index event; however, this rate decreases during the follow-up period. The proportion of patients achieving the target LDL level decreases to 35% [18]. Dyslipidemia and metabolic dysfunction of adipocytes and hepatocytes may contribute to a fourfold higher incidence of NAFLD in obese individuals compared to their non-obese counterparts [19]. The close relationship of all these metabolic syndrome-related diseases and the predisposition of metabolic syndrome to atherosclerotic heart disease supports the association between high NAFLD score and CAD in our study. Numerous population-based studies have identified NAFLD as an independent cardiovascular risk factor and an independent predictor for the 10-year risk of cardiovascular disease. Cardiovascular disease constitutes the predominant cause of mortality (40%) in patients with NAFLD [20]. NAFLD affects the coronary arteries through atherosclerosis and may also lead to cardiomyopathy secondary to diastolic dysfunction and hypertrophy, valve calcification due to valve sclerosis, and cardiac arrhythmias, particularly atrial fibrillation [21].

Its silent course, high prevalence rate, and close association with cardiovascular events require more attention to NAFLD. The disease is usually diagnosed by incidentally detected elevated liver enzyme levels or fatty liver on imaging. Since cardiovascular involvement is the most common cause of death in NAFLD, cardiovascular risk evaluation should be the standard assessment for the disease. The link between NAFLD and CAD raises the hypothesis that evaluation of NAFLD should be considered in the cardiovascular risk assessment of the general population [22]. Although liver biopsy is the gold standard for diagnosis, it is an invasive method with serious complications. This issue has led to the implementation of alternative methods, such as clinical scoring. In particular, European guidelines recommend these scoring methods to all patients with NAFLD [23]. The NFS differs from other prognostic scores in that it was developed from patients with biopsy-proven NAFLD diagnoses. The score predicts NAFLD with a sensitivity of 84% and specificity of 69%. The negative predictive value (81–98%) is significant, particularly for advanced fibrosis [10,11].

To reduce cardiovascular morbidity and mortality, it is recommended for NAFLD patients to undergo evaluation for cardiovascular risk every 1–2 years [8]. However, current scoring systems may be inadequate in determining the cardiovascular risk. A Korean population study demonstrated an association between fatty liver index and Framingham 10-year cardiovascular risk [24]. Our data represent that the significant increase in the fQRS-T angle, an indicator of high cardiovascular risk, in the high NFS group is consistent with previous results. In a study conducted by Wu et al. on 18,151 patients, they determined that apolipoprotein B and lipoprotein a were superior predictors of disease progression compared to the Framingham Risk Score alone. The study reported that the Framingham Risk Score was underestimated in predicting cardiovascular disease in NAFLD patients [25]. In our research, LDL was an independent predictor of high NFS in multivariate analysis, consistent with the dyslipidemia–NAFLD relationship demonstrated by Wu.

Patients with NAFLD are asymptomatic in the early stages; however, even early-stage fibrosis is associated with increased mortality, underscoring the importance of early diagnosis. Unfortunately, no signs or symptoms reliably indicate the presence of early-stage NAFLD. Despite the risk of CAD, no effective screening test has been demonstrated in patients with asymptomatic NAFLD [26]. The FQRS-T angle is a repolarization anomaly that has prognostic value. It can be easily evaluated, particularly in ischemia, and is displayed early on the ECG. An increased fQRS-T angle and NFS are associated with poor prognosis. Clinical evaluation of these two prognostic indicators together is likely to be more useful than determining risk alone. Our research demonstrates that the independent relationship between the fQRS-T angle and NAFLD supports this hypothesis.

Limitations: The research was carried out at a single facility with a limited patient population. The observational, cross-sectional nature of the study precludes the establishment of a cause-and-effect relationship between the fQRS-T angle and NFS. It is known that the spatial QRS-T angle is superior to the frontal QRS-T angle in assessing cardiovascular risk. Another limitation is that the spatial QRS-T angle was not included in the study. Non-invasive scores, while practical, exhibit significant variability and may lack precision in accurately identifying liver fibrosis and assessing its stage. Although NFS is a reliable scoring tool in the predictive scoring of NAFLD, an imaging method superior to scoring tools such as FibroScan or liver biopsy in detecting the intensity and staging of liver fibrosis was not used. Future prospective and large-scale studies examining the correlation between FibroScan or liver biopsy-confirmed NAFLD and the frontal QRS-T angle, or preferably, the spatial QRS-T angle, will certainly enhance our knowledge about the research topic. The temporal relationship between CAD and NAFLD could not be evaluated due to the study’s omission of disease duration.

## 5. Conclusions

The study indicated an independent association between the fQRS-T angle and NFS, a NAFLD risk scoring tool, in stable angina pectoris patients. Large-scale prospective studies evaluating both cardiovascular prognosis indicators, easily derived from ECG, laboratory, and clinical parameters, will shed light on this issue.

## Figures and Tables

**Table 1:** Demographic and medical data of the patients.

Variable	NAFLDH < 0.67 (N = 155)	NAFLDH ≥ 0.67 (N = 22)	*p*
Demographic Data			
Gender (Male) n (%)	114 (73.5)	15 (68.2)	0.382
Age (Year)	55.8 ± 12.4	71.6 ± 7.4	<0.001
Hypertension n (%)	76 (49)	17 (77.3)	0.011
Dyslipidemia n (%)	42 (27.1)	9 (40)	0.139
Diabetes Mellitus n (%)	33 (21.3)	12 (54.5)	0.002
Previous Stent n (%)	53 (34.2)	10 (45.5)	0.305
Coronary artery disease n (%)	96 (61.9)	21 (95.5)	0.002
Body mass index (kg/m^2^)	28.9 ± 4.5	30.3 ± 4.7	0.173
Current Smoking n (%)	68 (43.9)	7 (31.8)	0.201
Medication			
Aspirin n (%)	62 (40)	12 (54)	0.144
P2Y12 Inhibitors n (%)	4 (2.6)	3 (13.6)	0.042
ACEI n (%)	35 (22.6)	10 (45.5)	0.024
ARB (%)	31 (20)	4 (18.2)	0.552
Beta Blocker n (%)	42 (27.1)	11 (50)	0.029
Calcium channel blocker n (%)	13 (8.4)	4 (18.2)	0.142
Statin n (%)	24 (15.5)	6 (27.3)	0.168
Oral antidiabetic n (%)	28 (18.1)	10 (45.5)	0.006
Insulin n (%)	7 (4.5)	2 (9.1)	0.310
Laboratory Data			
Glucose (mg/dL)	111.9 ± 35	147 ± 74	<0.001
Se Creatinine (mg/dL)	0.91 ± 0.26	1.02 ± 0.23	0.075
White blood cell (10^3^/μL)	8.4 ± 2.33	9.1 ± 3.1	0.174
Neutrophil (10^3^/μL)	5.4 ± 2.1	6.6 ± 2.9	0.021
Hemoglobin (gr/dL)	14.1 ± 1.8	13.2 ± 2.1	0.034
Platelet (10^3^/μL)	253 ± 71	187 ± 37	<0.001
Aspartate aminotransferase (IU/L)	24.5 ± 9.9	42.5 ± 30.8	<0.001
Alanine aminotransferase (IU/L)	23.9 ± 12.6	21.4 ± 11.1	0.391
Albumin (g/dL)	4.2 ± 0.3	3.83 ± 0.82	<0.001
Total cholesterol (mg/dL)	207 ± 48	177 ± 37	0.006
Low-density lipoprotein (mg/dL)	134 ± 39	109 ± 34	0.005
High-density lipoprotein (mg/dL)	46 ± 12	44 ± 10.6	0.344
Triglyceride (mg/dL)	154.7 ± 74	133.4 ± 60	0.200
Electrocardiographic Data			
Pathologic Q n (%)	31(20)	8 (36.4)	0.077
Fragmented QRS n (%)	44 (28.6)	9 (40.9)	0.175
QRS Time (ms)	96.3 ± 14.3	105.1 ± 17.7	0.010
P-Max (ms)	103.02 ± 6.5	107.1 ± 11.2	0.019
P-Min (ms)	57.2 ± 5.4	59.7 ± 9.6	0.086
P-Dispersion (ms)	45.7 ± 4.1	47.7 ± 4.7	0.048
QT Interval (ms)	391 ± 34	405 ± 43	0.106
QTc Interval (ms)	416 ± 26	431 ± 31	0.018
R Peak Time (ms)	35.1 ± 11	40.8 ± 13.1	0.047
Frontal QRS-T Angle (°)	24 (14–92)	118 (78–149)	<0.001
Frontal QRS-T Angle (Log) (°)	3.2 ± 1.1	4.4 ± 0.73	<0.001

ACEI: Angiotensin-converting enzyme inhibitors, ARB: Angiotensin receptor blockers.

**Table 2:** Univariate and multivariate logistic regression analyses.

	Univariate			Multivariate		
Variable	OR	95% CI	*p*	OR	95% CI	*p*
Hypertension	3.354	1.242–10.02	0.018			
Coronary artery disease	12.91	1.691–98.84	0.014			
Neutrophil	1.231	1.027–1.475	0.024			
Hemoglobin	0.791	0.635–0.987	0.038			
Total cholesterol	0.986	0.975–0.996	0.007			
Low-density lipoprotein	0.982	0.969–0.995	0.007	0.984	0.970–0.998	0.024
QRS Time	1.032	1.006–1.059	0.015			
P-Max	1.064	1.007–1.124	0.026			
P-Dispersion	1.108	0.999–1.228	0.052			
QTc Interval	1.019	1.003–1.035	0.020			
R Peak Time	1.032	0.999–1.067	0.055			
Frontal QRS-T Angle (Log)	3.314	1.840–5.699	<0.001	3.472	1.886–6.395	<0.001 0.002

## Data Availability

The data presented in this study are available upon reasonable request to the corresponding author.
